# Requirement of γ-Aminobutyric Acid Chemotaxis for Virulence of *Pseudomonas syringae* pv. *tabaci* 6605

**DOI:** 10.1264/jsme2.ME20114

**Published:** 2020-11-07

**Authors:** Stephany Angelia Tumewu, Hidenori Matsui, Mikihiro Yamamoto, Yoshiteru Noutoshi, Kazuhiro Toyoda, Yuki Ichinose

**Affiliations:** 1 Graduate School of Environmental and Life Science, Okayama University, Tsushima-naka 1–1–1, Kita-ku, Okayama 700–8530, Japan

**Keywords:** bacterial virulence, chemotaxis, GABA, plant-microbe interaction, *Pseudomonas*

## Abstract

γ-Aminobutyric acid (GABA) is a widely distributed non-proteinogenic amino acid that accumulates in plants under biotic and abiotic stress conditions. Recent studies suggested that GABA also functions as an intracellular signaling molecule in plants and in signals mediating interactions between plants and phytopathogenic bacteria. However, the molecular mechanisms underlying GABA responses to bacterial pathogens remain unknown. In the present study, a GABA receptor, named McpG, was conserved in the highly motile plant-pathogenic bacteria *Pseudomonas syringae* pv. *tabaci* 6605 (*Pta*6605). We generated a deletion mutant of McpG to further investigate its involvement in GABA chemotaxis using quantitative capillary and qualitative plate assays. The wild-type strain of *Pta*6605 was attracted to GABA, while the Δ*mcpG* mutant abolished chemotaxis to 10‍ ‍mM GABA. However, Δ*mcpG* retained chemotaxis to proteinogenic amino acids and succinic semialdehyde, a structural analog of GABA. Furthermore, Δ*mcpG* was unable to effectively induce disease on host tobacco plants in three plant inoculation assays: flood, dip, and infiltration inoculations. These results revealed that the GABA sensing of *Pta*6605 is important for the interaction of *Pta*6605 with its host tobacco plant.

Phytopathogenic bacteria encounter harsh environmental conditions as soon as they arrive at the surface of their host, which may decrease their chance of survival. Therefore, these bacteria need to develop a system that enables them to sense environmental stimuli and respond accordingly. Most phytopathogenic bacteria detect and use signals coming from a host plant to their advantage by moving towards nutrient sources or away from potential danger. These sophisticated behaviors are known as chemotaxis (Adler, 1966; Sourjik and Wingreen, 2012). Chemoreceptors are methyl-accepting chemotaxis proteins (MCPs) located outside the cell membrane and in the cytosol. When MCPs perceive environmental compounds, they transduce the signals to their cytoplasmic signaling domain (SD). Bacteria possess 5 to 60 *mcp* genes encoding MCPs (Alexandre *et al.*, 2004). Methylation helices, parts of SD, are important for adaptation with contributions from methyltransferase CheR and methylesterase CheB. SD then relays signals to the coupling protein CheW and to two component systems, CheA and CheY. Phosphorylated CheY affects the direction of flagellar motor rotation (clockwise/tumble or counter-clockwise/swim) (Bi and Lai, 2015; Huang *et al.*, 2019).

Some MCPs in plant-associated bacteria have been successfully characterized. MCPs for amino acids and organic acids have been identified in *Pseudomonas fluorescens* Pf0-1, a plant-protective bacterium (Oku *et al.*, 2012, 2014). Furthermore, the McpS, McpQ, and McpG of *Pseudomonas putida* KT2440 are chemoreceptors for tricarboxylic cycle intermediates (Lacal *et al.*, 2010), citrate (Martin-Mora *et al.*, 2016b), and γ-aminobutyric acid (GABA) (Reyes-Darias *et al.*, 2015), respectively. The identification of bacterial chemoreceptors has expanded to bacterial species with different lifestyles.

Pathogenic bacteria employ chemotaxis to enhance pathogenicity and virulence (Matilla and Krell, 2018). In *Pseudomonas aeruginosa*, an animal pathogen and major model pathogen in the study of chemotaxis, several chemoreceptors were identified for proteinogenic amino acids (Kato *et al.*, 2008), GABA (Rico-Jiménez *et al.*, 2013), and α-ketoglutarate (Martin-Mora* et al.*, 2016a). Furthermore, specific chemoreceptors for malate, amino acids, and boric acid, which are considered to be important for the mediation of chemotaxis in plant infection, were identified in the tomato bacterial wilt pathogen, *Ralstonia pseudosolanacearum* (Hida *et al.*, 2015, 2017). As previously reported, *Pseudomonas syringae* moves towards wounds or natural openings in host plants and induces disease, indicating chemotactic behavior towards molecules leaving these openings (Melotto *et al.*, 2006). The requirement for adaptation for survival may force pathogens to evolve multiple chemoreceptor proteins, MCPs. In *Escherichia coli* strain K-12, which possesses only five chemoreceptors, the specificity of ligand recognition within the ligand-binding domain (LBD) has been extensively characterized (Bi and Lai, 2015). Very recently, PscA of *P. syringae* pv. *tomato* DC3000 (*Pto*DC3000) was identified as a chemoreceptor for L-Asp, L-Glu, and D-Asp, and the virulence of the *pscA* mutant was reduced (Cerna-Vargas *et al.*, 2019). However, the majority of chemoreceptor functions have not yet been elucidated due to the difficulties associated with functionally analyzing up to 50‍ ‍MCPs.

PctC and McpG, chemoreceptors for GABA, were recently reported in *P. aeruginosa* PAO1 (Rico-Jiménez *et al.*, 2013) and saprophytic *P. putida* KT2440 (Reyes-Darias *et al.*, 2015), respectively, supporting the universal importance of GABA as a signaling molecule. Furthermore, the PscC of the kiwifruit pathogen *P. syringae* pv. *actinidiae* NZ-V13 (*Psa*NZ-V13) has been reported as a potential MCP for GABA based on isothermal titration calorimetric findings showing that GABA bound the LBD of PscC (McKellar *et al.*, 2015). However, a chemotaxis assay and virulence test were not performed on *Psa*. Thus, the chemoreceptors of GABA in plant-pathogenic bacteria have not yet been fully characterized. Since GABA controls multiple aspects of microbe-plant interactions, the identification of GABA chemoreceptors in plant-pathogenic bacteria may provide a more detailed understanding of its physiological relevance in plant-pathogen interactions.

Plants synthesize various chemical compounds in response to stress conditions. One of the chemicals produced is GABA, a non-proteinogenic amino acid that is widely distributed in prokaryotic and eukaryotic organisms (Fig. 1A) (Dhakal *et al.*, 2012; Seifikalhor *et al.*, 2019). GABA is produced inside plant cells and regulates plant growth (Tarkowski *et al.*, 2020). It is also a plant defense modulator that plays an important role during environmental stress (Tarkowski *et al.*, 2020). In plants, GABA is synthesized via the GABA shunt in which glutamate decarboxylase (GAD) converts glutamate into GABA (Michaeli and Fromm, 2015). GABA accumulates, particularly in plant apoplasts, when plants encounter biotic (*i.e.*, pathogen invasion) (Rico and Preston, 2008) or abiotic stress (*i.e.*, mechanical damage) (Mei *et al.*, 2016). Furthermore, GABA levels have been shown to affect stomatal closure (Mekonnen *et al.*, 2016).

In *Agrobacterium tumefaciens*, GABA mediates quorum sensing by stimulating the inactivation of quorum-sensing molecules (Chevrot *et al.*, 2006). Furthermore, in *A. tumefaciens*, reductions in GABA concentrations by the introduction of *gabT*, a gene encoding GABA transaminase, enhanced the transfer efficiency of Ti plasmids to tomato plants (Nonaka *et al.*, 2017), and elevated GABA levels in transgenic tobacco plants reduced sensitivity to *A. tumefaciens* infection (Chevrot *et al.*, 2006). On the other hand, the accumulation of GABA in *Pseudomonas protegens* appeared to enhance its colonization ability (Takeuchi, 2018). GABA has been found to reduce the ability of *Pto*DC3000 to infect host plants (Park *et al.*, 2010; Chatnaparat *et al.*, 2015; McCraw *et al.*, 2016). Another study demonstrated that *Phaseolus vulgaris* accumulated extracellular GABA during incompatible interactions with *P. syringae* pv. *phaseolicola* (O’Leary *et al.*, 2016). Therefore, GABA functions may vary specifically among species; however, it generally serves to mediate inter-kingdom communication in both cases.

In the present study, the highly chemotactic *Pseudomonas syringae* pv. *tabaci* 6605 (*Pta*6605), which possesses approximately 50 MCPs, exhibited strong chemotaxis towards GABA and was found to possess a GABA chemoreceptor. As previously reported, chemotaxis is indispensable for *Pta*6605 virulence (unpublished data). Therefore, we hypothesized that sensing GABA may modulate *Pta*6605 interactions with its host plant. Using a deletion mutant of the predicted GABA receptors, different virulence assay methods were conducted to investigate the importance of GABA chemotaxis for *Pta*6605.

## Materials and Methods

### Bacterial strains and plasmids

Bacterial strains and plasmids are listed in Table 1. *Pta*6605 strains were grown in King’s B (KB) medium supplemented with 50‍ ‍μg mL^–1^ nalidixic acid (Nal) at 27°C (King *et al.*, 1954; Taguchi *et al.*, 2006). *E. coli* strains were grown in Luria Bertani (LB) medium supplemented with appropriate antibiotics (50‍ ‍μg mL^–1^) at 37°C.

### Construction of predicted GABA receptor mutants in *Pta*6605

Three deletion mutants were generated for the locus tags, A3SK_RS0126685, A3SK_RS0106980, and A3SK_RS0112400 in *Pta*6605. Since they are not yet annotated, we hereafter use the tentative names RS26685, RS06980, and RS12400, respectively, for each locus tag. Several primer pairs (listed in Table 2) were used to amplify the corresponding regions with surrounding sequences from *Pta*6605 genomic DNA. The resulting PCR products were inserted into a pGEM T-Easy Vector (Promega), and inverse PCR was performed with appropriate primer pairs to delete each open reading frame of *mcp* (Fig. S1). Following *Xba*I digestion and self-ligation, mutated DNA fragments were subcloned into pK18*mobsacB* via a *Not*I site (Schäfer *et al.*, 1994) and transformed into *E. coli* S17-1 for conjugation and subsequent homologous recombination with *Pta*6605 wild-type (WT), as previously reported (Ichinose *et al.*, 2020). Colony PCR of the conjugants was performed using the respective primer pairs for confirmation. Deletion mutants were designated as ΔRS26685 (Δ*mcpG*), ΔRS06980, and ΔRS12400.

### Complementation of *Pta*6605 Δ*mcpG*

To investigate the ability of *mcpG* to restore the phenotype of Δ*mcpG*, complementation strains were constructed by introducing the full length of the *mcpG* gene along with its native promoter in the expression vector pDSK519 (Keen *et al.*, 1988) at *Not*I sites. The plasmid constructed was transformed into *E. coli* S17-1 and introduced into Δ*mcpG* by conjugation. Conjugants were selected with the Nal- and kanamycin-containing KB plates.

### Chemotaxis assays

Quantitative chemotaxis assays were performed using the microtiter plate multi-capillaries method (Reyes-Darias *et al.*, 2016) with minor modifications. Bacteria were cultured overnight in 3‍ ‍mL LB with 10‍ ‍mM MgCl_2_, washed, and resuspended in 3‍ ‍mL minimal medium (MM: 50‍ ‍mM potassium phosphate, 7.6‍ ‍mM [NH_4_]_2_SO_4_, 1.7‍ ‍mM MgCl_2_, and 1.7‍ ‍mM NaCl). One hundred and fifty microliters of the bacterial suspension was then inoculated into 3‍ ‍mL of fresh MM supplemented with 10‍ ‍mM of mannitol and fructose (MMMF) for a further 5-h incubation. Bacterial cells were washed twice and resuspended with 10‍ ‍mM HEPES (pH 7.4) to OD_600_ of 0.05. Capillary preparation was conducted as follows: one end of a 5-μL capillary (Drummond Scientific Company) was sealed by heating with a flame. The capillary body was dipped into a potential chemoattractant solution or 10‍ ‍mM HEPES buffer as a control. A rubber collar was used to support the capillary during the assay. Two hundred microliters of the bacterial suspension was pipetted into three wells of a round-bottomed Falcon^®^ microtiter plate (Corning). The chemical-filled capillary was dipped into the wells and incubated at 27°C for 30‍ ‍min. After rinsing with sterile distilled water, 5‍ ‍μL of the capillary content was squirted into 45‍ ‍μL of 0.9% NaCl. A series of 10-fold dilutions was performed, and 10‍ ‍μL of the diluted bacterial suspension was plated onto a KB plate with 50‍ ‍μg mL^–1^ Nal. After a 2-day incubation at 27°C, the number of colonies was counted.

A qualitative chemotaxis assay was performed by observing the ability of WT and mutant strains to swim on MM plates supplemented with 1‍ ‍mM of GABA (Wako Pure Chemicals) and 0.25% Bacto agar (Difco). Briefly, overnight bacterial cultures in LB with 10‍ ‍mM MgCl_2_ were washed and resuspended in liquid MM to OD_600_ of 0.1. Three microliters of the bacterial suspension was dropped carefully on the center of a freshly made chemotaxis swimming plate. Photographs were taken after the incubation at 23°C for 3 d.

### Surface motility assay

Surface motility assays measured swimming and swarming abilities on semi-solid agar plates. Bacteria cultured overnight in 3‍ ‍mL LB with 10‍ ‍mM MgCl_2_ were washed and resuspended in 10‍ ‍mM MgSO_4_ to an OD_600_ of 0.1. Three microliters of the bacterial suspension was spotted onto the center of the plate. Swimming plates (MMMF with 0.25% agar) were incubated at 23°C, while swarming plates (0.5% peptone, 0.3% yeast extract, and 0.45% agar) were incubated at 27°C. Photographs were taken on the 2‍ ‍d for the swarming assay and on the 3‍ ‍d for the swimming assay after the inoculation.

### Bacterial growth *in vitro*

Bacteria were cultured overnight in 3‍ ‍mL KB with 50‍ ‍μg mL^–1^ Nal, washed with KB or MMMF, and resuspended in the same fresh medium to a starting OD_595_ of 0.1. Each bacterial suspension was pipetted into a flat-bottomed 96-well microtiter plate and incubated at 27°C with shaking. Bacterial cell density (OD_595_) was measured using an iMark^TM^ Microplate Absorbance Reader (Bio-Rad Laboratories) every 2 h to generate growth curves.

### Virulence assays and *in planta* bacterial populations

To thoroughly assess the involvement of McpG in *Pta*6605 virulence, three plant inoculation assays: flood, dip, and infiltration inoculations, were performed. A flood inoculation method (Ishiga *et al.*, 2011) was modified for tobacco seedlings. Sterilized tobacco seeds (*Nicotiana tabacum* L. var. Xanthi NC) were sown on Murashige-Skoog (MS) 0.8% agar plates containing 1% sucrose and vitamin stock solution (thiamin hydrochloride 3‍ ‍mg L^–1^, nicotinic acid 5‍ ‍mg L^–1^, and pyridoxine hydrochloride 0.5‍ ‍mg L^–1^). Sown plates were incubated at 28°C under 16-h light/8-h dark conditions. After 2‍ ‍weeks, 8 seedlings were transplanted to one MS 0.8% agar plate containing 0.1% sucrose and vitamin stock solution and further incubated for 2 d. To prepare the inoculum, bacteria cultured overnight in LB medium with 10‍ ‍mM MgCl_2_ were washed and adjusted to OD_600_=0.004 (8×10^6^ colony-forming units [CFU] mL^–1^) with 10‍ ‍mM MgSO_4_ (approx. 30‍ ‍mL) and 0.025% (v/v) Silwet L-77 (OSI Specialties). The inoculum was flooded onto the seedlings and swirled to spread it evenly. After decanting the bacterial suspension, the plate was air-dried on a clean bench for 15‍ ‍min. Seedlings on the plates were incubated under 16-h light/8-h dark conditions at 22°C. To count bacterial populations, leaf disks were punched out using a disposable biopsy hole punch and then ground with a mortar and pestle. Leaf disks were collected 3 h post inoculation (hpi), homogenates were mixed with 1‍ ‍mL of sterile water, and 100‍ ‍μL was then spread on KB with Nal plates. After 10-fold serial dilutions 3‍ ‍d post inoculation (dpi), 10‍ ‍μL was dropped on KB with Nal plates. After an incubation at 27°C for 2‍ ‍d, the bacterial population was measured.

The dip inoculation method was also performed using detached tobacco leaves (Taguchi and Ichinose, 2011). The inoculum preparation was basically the same as that for the flood inoculation method, except that the inoculum density was adjusted to OD_600_ of 0.1 (the approximate density of bacteria was 2×10^8^ CFU mL^–1^) with 0.04% (v/v) Silwet L-77 for this method. In this method, the detached leaves of 8-week-old tobacco plants were dipped into the bacterial suspension for 2‍ ‍min and placed in a tray covered with plastic wrap. Cut petioles were wrapped with water-soaked cotton. The tray was incubated under 16-h light/8-h dark conditions at 22°C for 10 d. The same inoculum preparation was also performed for the infiltration method. Eight-week-old tobacco leaves (attached leaves) were infiltrated by bacterial cells at a density of 2×10^5^ CFU mL^–1^ using a needleless syringe. Inoculated tobacco plants were incubated in a domed tray at 22°C with a long-day photoperiod (16-h light/8-h dark) for 14 d.

### Data analyses

The bacterial population counts of chemotaxis and virulence assays were expressed as means with standard errors. One-way/two-way ANOVA followed by Dunnett’s or Tukey’s highly significant difference tests were performed using GraphPad Prism ver. 8 (GraphPad Software). *P*<0.05 was considered to be significant.

## Results

### *Pta*6605 was predicted to have three GABA receptor homologs

**Fig. 1.**
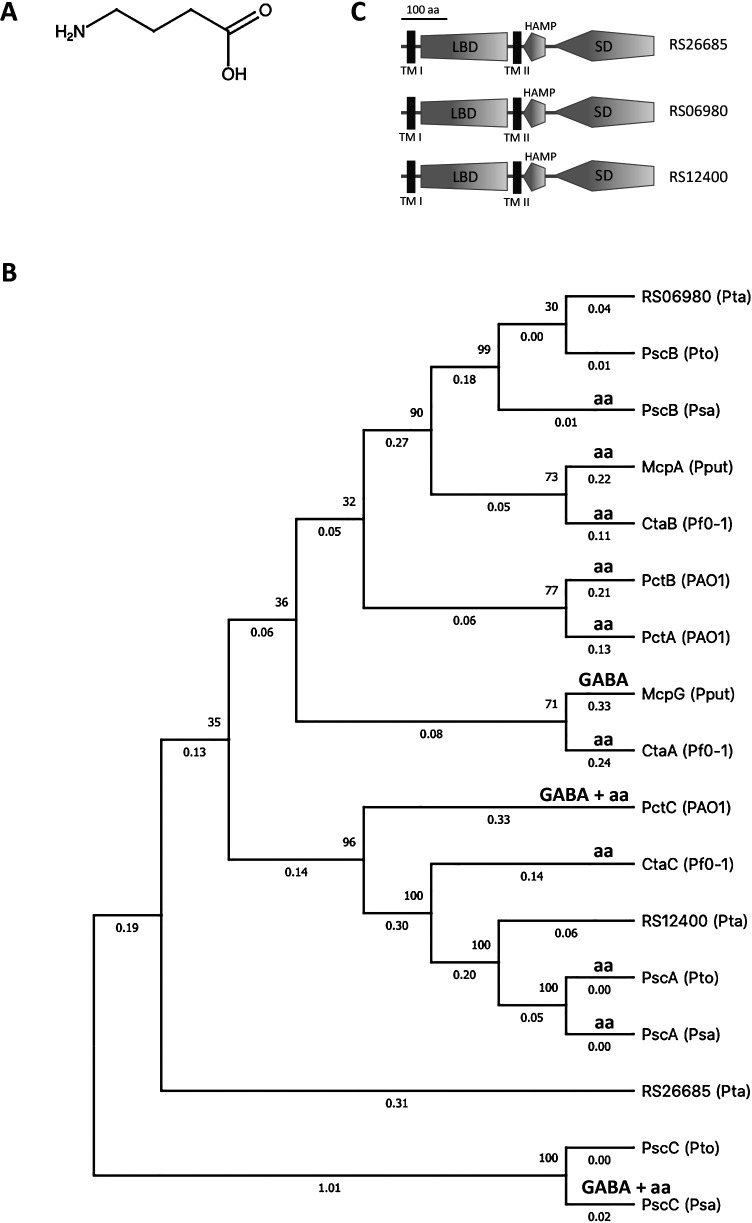
GABA structure (A) and maximum likelihood tree based on ligand-binding domains (LBDs) of amino acid (aa) receptors and their homologs in *Pseudomonas* species (B). Phylogenetic tree of amino acid sequences of the LBD of McpG and McpA, a GABA and amino acid receptor of *Pseudomonas putida* KT2440; PctC, a GABA receptor and PctA and PctB, amino acid receptors of *Pseudomonas aeruginosa* PAO1; CtaABC, amino acid receptors of *Pseudomonas fluorescens* Pf0-1; PscABC, potential receptors for amino acids and GABA of *Psa*NZ-V13 and amino acid receptor and its paralogs of *Pto*DC3000; RS26685, RS12400, and RS06980, predicted GABA and amino acid receptors of *Pta*6605. Corresponding ligands are indicated by bold letters (**aa** and **GABA**). Branch length and bootstrap values are indicated on the tree. The tree was generated using MEGA version X software. Schematic structure of three MCP proteins of *Pta*6605 (C). Transmembrane domains (TMI and TMII), ligand-binding domains (LBD), *h*istidine kinase, *a*denyl cyclase, *m*ethyl-accepting chemotaxis proteins and *p*hosphatase (HAMP) domains, signaling domains (SD), and the scale of 100 amino acids are indicated.

An MCP typically has LBD, transmembrane domains (TMD), a cytoplasmic *h*istidine kinase, *a*denyl cyclase, *m*ethyl-accepting chemotaxis proteins, and *p*hosphatase (HAMP) domain, and SD (Ud-Din and Roujeinikova, 2017). A BLAST search with the LBD of McpG (PP1371), a GABA receptor in *P. putida* KT2440 (Reyes-Darias *et al.*, 2015) revealed three GABA chemoreceptor homologs in *Pta*6605 with 64.9% (RS26685), 59.9% (RS06980), and 59.4% (RS12400) amino acid sequence identities. The amino acid sequences of the LBDs of GABA chemoreceptors in *P. aeruginosa* PAO1 (PctC and its paralogs PctA and PctB) (Taguchi *et al.*, 1997) and *P. putida* KT2440 (McpG) (Reyes-Darias *et al.*, 2015), amino acid receptors in *P. fluorescens* Pf0-1 (CtaA, CtaB, and CtaC) (Oku *et al.*, 2012), amino acid receptors in *Psa*NZ-V13 (PscA and PscB) (McKellar *et al.*, 2015), a potential GABA receptor in *Psa*NZ-V13 (PscC) (McKellar *et al.*, 2015), amino acid receptors in *Pto*DC3000 (PscA and its paralogs PscB and PscC) (Cerna-Vargas *et al.*, 2019), and three suspected GABA receptor homologs in *Pta*6605 were aligned (Fig. S2) to generate a phylogenetic tree (Fig. 1B). RS12400 was clustered together with the PscA of *Pto*DC3000 and *Psa*NZ-V13, and the RS06980 of *Pta*6605 was clustered together with the PscB of *Pto*DC3000 and *Psa*NZ-V13. However, the RS26685 of *Pta*6605 appeared to cluster separately from the LBDs of other MCPs, suggesting that any of the three MCPs in *Pta*6605 were chemoreceptors for GABA in *Pta*6605.

MCPs may be classified into seven topology types (Ia, Ib, II, IIIm, IIIc, IVa, and IVb) based on the number of TMD, the presence or absence of LBD, and its localization (Ud-Din and Roujeinikova, 2017). Three GABA receptor homologs in *Pta*6605 belong to type Ia MCP. Type Ia MCPs consist of an N-terminal TMD followed by a periplasmic LBD, a second TMD, HAMP, and a C-terminal cytoplasmic SD (Fig. 1C). We confirmed that all MCPs for GABA and amino acids listed in Fig. 1B belonged to type Ia.

### Identification of McpG as a GABA chemoreceptor in *Pta*6605

**Fig. 2.**
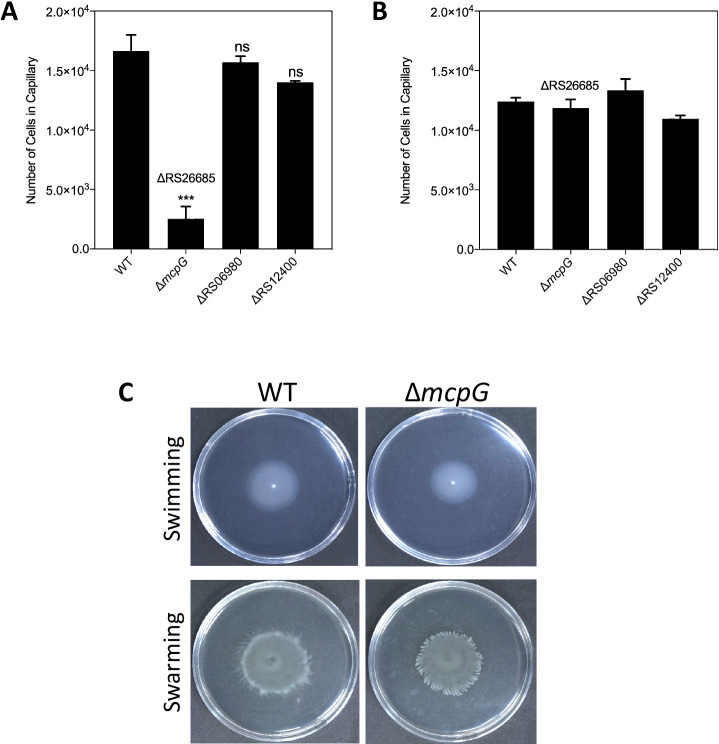
Screening for GABA receptors. Chemotaxis to 10‍ ‍mM GABA (A) and 1% yeast extract (B). Asterisks indicate a significant difference from the WT strain (ns: not significant; *** *P*<0.001 by a one-way ANOVA followed by Dunnett’s multiple comparisons test). Error bars represent standard errors from 2 independent experiments conducted in triplicate. (C) Surface motility assay of WT and Δ*mcpG*. A swarming assay on SWM plates with 0.45% agar at 27°C for 2‍ ‍d and a swimming assay on MMMF plates with 0.25% agar at 23°C for 3 d. The photographs show representative results obtained from three independent experiments with two replicates.

Quantitative capillary assays were conducted to investigate how the mutation of each predicted GABA receptor affects *Pta*6605 chemotaxis to GABA. Quantified results showed significant reductions in ΔRS26685 chemotaxis to 10‍ ‍mM GABA, while ΔRS06980 and ΔRS12400 retained similar chemotaxis to GABA to WT (Fig. 2A). ΔRS26685 did not lose its general chemotaxis ability because ΔRS26685 was attracted to 1% yeast extract, similar to WT, ΔRS06980, and ΔRS12400 (Fig. 2B). These results indicated that RS26685 encodes the chemoreceptor for GABA in *Pta*6605, and we designated it as McpG.

### Δ*mcpG* swims and swarms on soft agar at the WT level

The surface motilities of WT and Δ*mcpG* (ΔRS26685) were examined using low-agar-concentration plates. Δ*mcpG* exhibited the same swimming and swarming abilities as WT (Fig. 2C). These results indicated that the inability of Δ*mcpG* to respond to GABA was not due to a lack of motility.

### Δ*mcpG* chemotaxis to GABA with different concentration and complementation tests

**Fig. 3.**
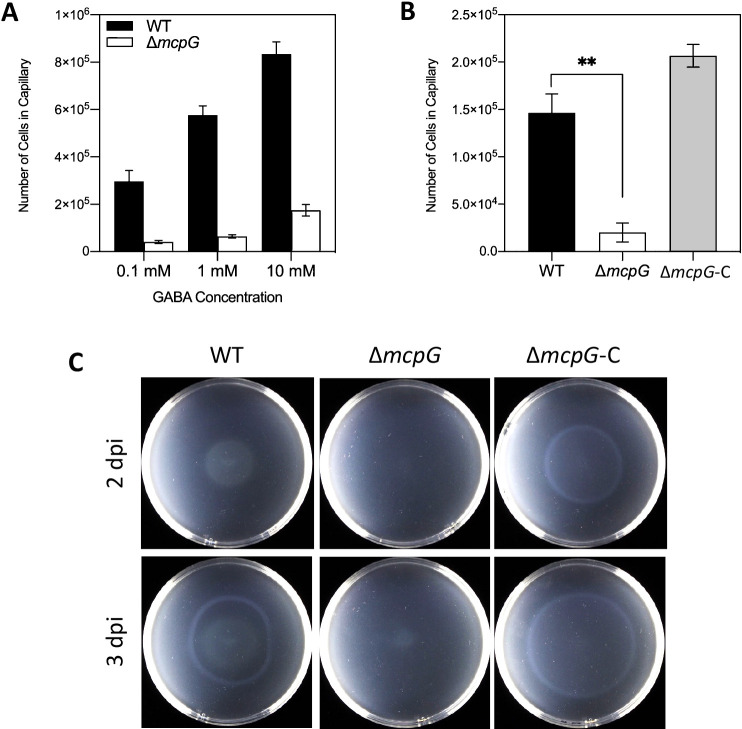
Chemotaxis assays. (A) Quantitative chemotaxis assay of WT and Δ*mcpG* towards different GABA concentrations. (B) Chemotaxis of Δ*mcpG* and its complementation strain (Δ*mcpG*-C) to 10‍ ‍mM GABA. Asterisks indicate significant differences from the WT strain (** *P*<0.01 by a one-way ANOVA followed by Dunnett’s Multiple Comparisons Test). Error bars represent standard errors from 2 independent experiments conducted in triplicate. (C) Qualitative chemotaxis plate assay on 0.25% agar minimal media supplemented with 1‍ ‍mM of GABA 2 and 3 dpi at 23°C. The photographs show representative results from three independent experiments with 2 plates each.

We investigated the chemotaxis of both WT and Δ*mcpG* to different concentrations of GABA. While WT responded to GABA in a dose-dependent manner, Δ*mcpG* showed significant reductions at all concentrations (Fig. 3A). Complementation by the introduction of full-length *mcpG* with its native promoter into Δ*mcpG* restored chemotaxis to GABA (Fig. 3B). We also qualitatively investigated Δ*mcpG* chemotaxis using 0.25% agar MM supplemented with 1‍ ‍mM GABA. Δ*mcpG* was unable to expand its growth from its inoculation point 2 or even 3 dpi, while its complemented and WT strains expanded at similar levels (Fig. 3C).

### McpG is the specific chemoreceptor for GABA

**Fig. 4.**
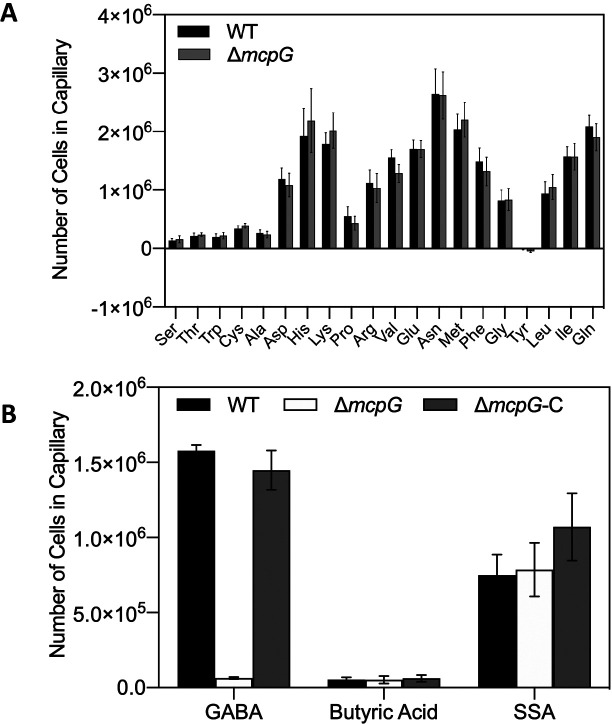
Quantitative chemotaxis assay to assess McpG specificity. (A) Chemotactic responses to 20 proteinogenic amino acids (1‍ ‍mM) and (B) chemotactic responses to GABA, butyric acid, and SSA (1‍ ‍mM). Error bars represent standard errors from 2 independent experiments conducted in triplicate.

We tested the chemotaxis ability of WT and Δ*mcpG* to 20 proteinogenic amino acids, butyric acid, and succinic semialdehyde (SSA), which share similar structures to GABA. Quantitative chemotaxis assay results demonstrated that *Pta*6605 was attracted to most proteinogenic amino acids, except for tyrosine. *Pta*6605 was also attracted to SSA, but not to butyric acid (Fig. 4). Furthermore, no significant differences were observed in chemotactic ability between WT and Δ*mcpG* to all amino acids and SSA. These results indicate that the McpG of *Pta*6605 is a specific chemoreceptor for GABA.

### Growth characteristics of Δ*mcpG*

The growth characteristics of WT and Δ*mcpG* were assessed to clarify whether mutations affect their growth and to confirm that the lack of chemotaxis in Δ*mcpG* was not due to a growth defect. Growth experiments were conducted in KB (nutrient rich) and MMMF liquid medium (Fig. S3A), and in MM supplemented with GABA as a sole source of carbon and nitrogen (Fig. S3B). The growth of Δ*mcpG* in KB and MMMF liquid medium was not significantly different from that of WT. In MM supplemented with GABA, although the growth of Δ*mcpG* appeared to be slower than that of WT, the difference was not significant at any time points, indicating that the phenotypic difference between them was not due to a growth defect.

### Δ*mcpG* had reduced virulence on host tobacco plants

**Fig. 5.**
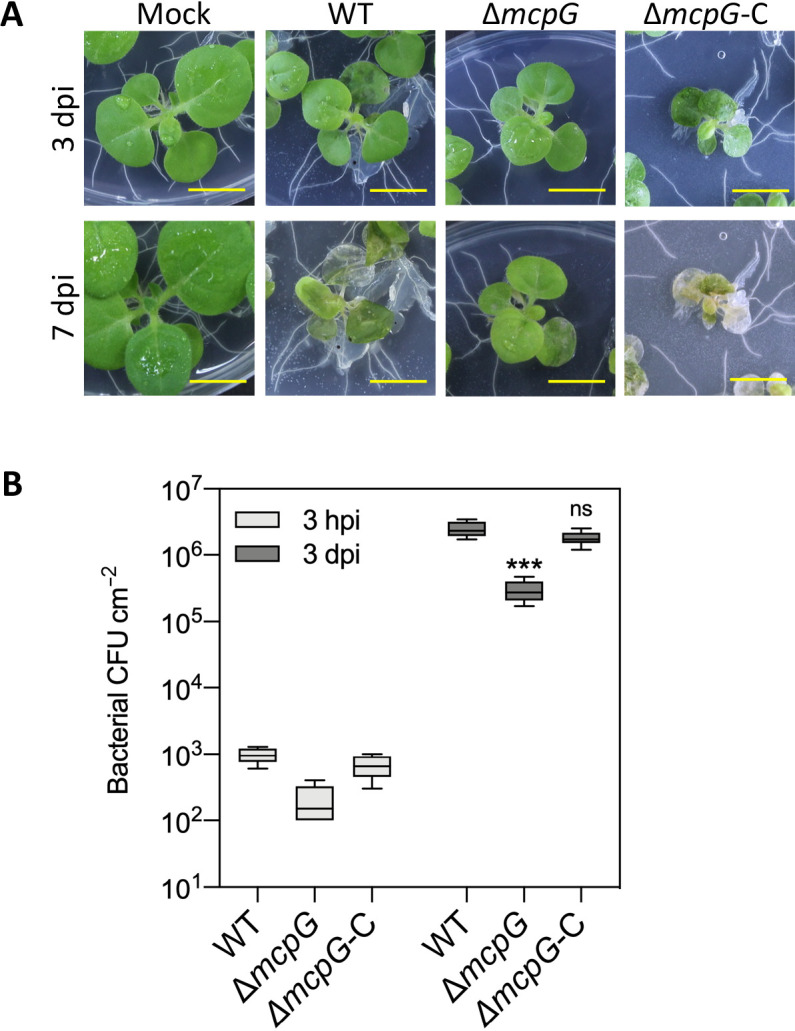
Inoculation experiments by the flood inoculation method. (A) Tobacco seedlings were inoculated by flooding 8×10^6^ CFU mL^–1^ of the bacterial suspension of each strain followed by an incubation at 22°C. Photographs taken 3 and 7 dpi show representative results from three independent experiments. (B) The bacterial population was counted 3 hpi and 3 dpi. Bars represent standard errors from two independent experiments. Bacterial CFUs for each strain in one experiment were pooled from 3 (3 hpi) or 4 (3 dpi) individuals. Asterisks indicate significant differences from the WT strain (* *P*<0.05; *** *P*<0.001 by a two-way ANOVA followed by Dunnett’s multiple comparisons test).

Plant inoculation assays were performed to elucidate how McpG-mediated chemotaxis affects *Pta*6605 virulence in host tobacco plants. Virulence was initially tested using the flood inoculation assay method. The photographs in Fig. 5A clearly show that Δ*mcpG*-inoculated seedlings still survived 7 dpi, but were smaller than those with the mock treatment. On the other hand, the complemented strain restored full virulence. We also counted bacterial populations inside the leaves. The number of bacteria recovered from Δ*mcpG*-inoculated leaves was significantly lower at both 3 hpi and 3 dpi (Fig. 5B), indicating that GABA sensing was required for the early stage of virulence in *Pta*6605.

**Fig. 6.**
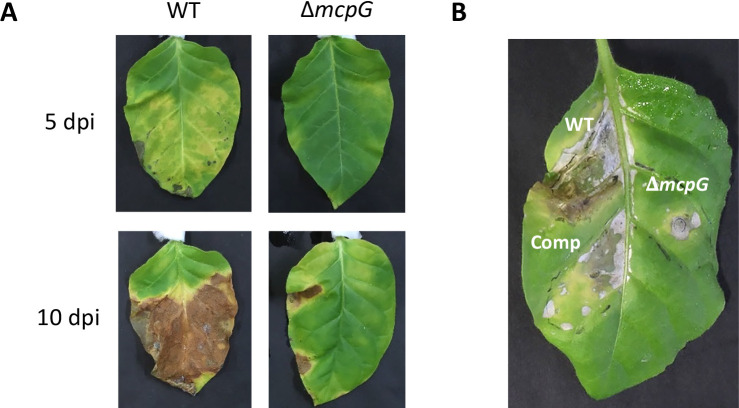
Inoculation test of WT and Δ*mcpG* strains by dip and infiltration methods. (A) Tobacco leaves were inoculated by dipping into 2×10^8^ CFU mL^–1^ of the bacterial suspension of each strain followed by an incubation at 22°C. Photographs taken 5 and 10 dpi show representative results from three independent experiments. (B) Tobacco leaves were infiltrated by 2×10^5^ CFU mL^–1^ of each strain and incubated at 22°C. Photographs taken 14 dpi show representative results from two independent experiments.

A second virulence test was performed using the dip inoculation method with cut leaves. Δ*mcpG* caused milder disease symptoms than the WT strain (Fig. 6A), indicating that McpG is also required for the development of disease symptoms in the dip inoculation method. The virulence of WT and Δ*mcpG* was also investigated by directly infiltrating bacterial cells into 5-week-old tobacco leaves. An area of the Δ*mcpG*-infiltrated leaf showed disease symptom progression, although it was not as robust as WT (Fig. 6B). However, the complemented strain caused more extensive symptoms than Δ*mcpG*.

## Discussion

RS26685 (McpG) was identified as a GABA chemoreceptor in *Pta*6605 based on the results of the quantitative chemotaxis assay (Fig. 2 and 3AB) and qualitative swimming plate assay (Fig. 3C). Normal chemotaxis to 1% yeast extract (Fig. 2B), surface motility (Fig. 2C), and growth speed (Fig. S3) proved that its lost ability to sense GABA was not due to impaired motility or a growth defect. Therefore, the reduction in the virulence of the Δ*mcpG* mutant was principally due to the loss of GABA chemotaxis. PscC is a potential GABA receptor of *Psa*NZ-V13; however, it has not yet been confirmed with a quantitative chemotaxis assay, and its requirement in the infection process remains unknown (McKellar *et al.*, 2015). This present study is the first to demonstrate that GABA chemotaxis is required for infections by phytopathogens. The identification of a GABA chemoreceptor in the plant pathogen *Pta*6605 widened the relevance of GABA chemotaxis, not only for attraction to neurotransmitters by the animal pathogen *P. aeruginosa* PAO1 and root colonization in non-pathogenic *P. putida* KT2440 (Reyes-Darias *et al.*, 2015).

Some GABA chemoreceptors recognize multiple amino acids. For example, the PctC of *P. aeruginosa* recognizes GABA, L-Pro, and L-His (Rico-Jiménez *et al.*, 2013), and the PscC of *Psa* binds GABA, L-Pro, and L-Ile (McKellar *et al.*, 2015). The McpG of *P. putida* is a specific GABA receptor (Reyes-Darias *et al.*, 2015). The McpG of *Pta*6605 also specifically mediates chemotaxis to GABA, similar to the McpG of *P. putida* KT2440, because the Δ*mcpG* mutant of *Pta*6605 strain was still attracted to 19 amino acids and SSA (Fig. 4). The presence of specific GABA receptors suggests the importance of GABA chemotaxis for *Pta*6605 infection.

The plant-pathogenic bacterium *Pta*6605 apparently possesses three paralogs of McpG, RS26685, RS06980, and RS12400, among approximately 50 MCPs. They may have a similar function to the PctA, PctB, and PctC of PAO1, PsaA, PsaB, and PsaC of *Psa*NZ-V13, and CtaA, CtaB, and CtaC of *P. fluorescens* Pf0-1 (Kato *et al.*, 2008; Oku *et al.*, 2012; Rico-Jiménez *et al.*, 2013; McKellar *et al.*, 2015), further suggesting that *Pta*6605 responds to environmental signals, such as GABA and amino acids.

Regarding the other two paralogs, both phylogenetic trees constructed from LBDs (Fig. 1B) and SDs (Fig. S4) suggested that RS06980 and RS12400 are MCPs involved in amino acid sensing based on close similarities to the amino acid chemoreceptors, McpA in *P. putida* KT2440 (Corral-Lugo *et al.*, 2016), Psc paralogs in *Psa*NZ-V13 and *Pto*DC3000 (McKellar *et al.*, 2015; Cerna-Vargas *et al.*, 2019), and Cta paralogs in *P. fluorescens* Pf0-1 (Oku *et al.*, 2012). However, the phylogenetic tree based on the sequence similarity of LBD suggests that the ability to sense GABA is not really reflected in the general sequence similarities of LBDs, thereby proving that the characterization of responsible chemoreceptors did not solely depend on sequences clustering from one or two strains of bacteria (Fig. 1B). Further studies are needed to clarify the functions of both chemoreceptor proteins.

Bacteria often exhibit chemotaxis to molecules that they may utilize as growth substrates. The majority of chemical compounds that bacteria are attracted to are carbon or nitrogen sources, such as amino acids, sugars, organic acids, hydrocarbons, and oxygen (Sampedro *et al.*, 2015). Previous findings obtained on the fungal pathogen in tomato, *Cladosporium fulvum* (Solomon and Oliver, 2002), animal pathogen *P. aeruginosa* (Rico-Jiménez *et al.*, 2013), and saprophytic plant root-colonizing bacterium, *P. putida* (Reyes-Darias *et al.*, 2015) further support the concept that GABA is of physiological relevance in host-microbe interactions.

Plant-related compounds are known to regulate virulence factors in addition to the attractants of chemotaxis (Leonard *et al.*, 2017). GABA is produced in plants infected by bacterial pathogens (Deeken *et al.*, 2006) and secreted to the apoplast and outer environment (Mei *et al.*, 2016). The *Pta*6605 GABA-sensing mutant, Δ*mcpG*, was shown to be significantly less virulent in host tobacco by the flood inoculation method (Fig. 5) and dip inoculation method using detached leaves (Fig. 6A). These results revealed the necessity of GABA chemotaxis in the early stage of infection when bacterial cells arrive on the leaf surface. Furthermore, Δ*mcpG* was less virulent than WT even with infiltration using a needleless syringe (Fig. 6B). This result suggests the involvement of GABA not only in early, but also in late infection stages because the virulence of Δ*mcpG* that entered plant apoplastic spaces by infiltration independent of chemotaxis was also reduced. GABA perception by *Pta*6605 via McpG may be required not only for chemotaxis, but also for the expression of virulence genes. Similarly, the *pscA* mutant of *Pto*DC3000 also impaired not only chemotaxis to L-Asp, L-‍Glu, and D-Asp, but also the regulation of some pathogenicity-related traits, such as biofilm formation, swarming motility, and the amount of c-di-GMP (Cerna-Vargas *et al.*, 2019). Therefore, some MPCs, including the McpG of *Pta*6605 and PscA in *Pto*DC3000, act as both chemoreceptors and regulators of pathogenicity.

Since GABA is widely distributed, its physiological relevance as a signaling compound appears to be high. The accumulation of intracellular and extracellular GABA in plants is induced by biotic and abiotic stresses, such as nutrient depletion, mechanical wounding, pathogen infection, and a lack of oxygen (Allan *et al.*, 2008; Reyes-Darias *et al.*, 2015; Mei *et al.*, 2016; Seifikalhor *et al.*, 2019). Furthermore, the expression level of type III secretion system (T3SS) genes in plant pathogenic bacteria is higher *in planta*, indicating that the presence of plant-derived signals is required for the expression of T3SS genes and full virulence (Rahme *et al.*, 1992; Tang *et al.*, 2006). Therefore, it is plausible for a plant pathogen, such as *Pta*6605, to have a specific chemoreceptor for GABA for fitness in host plant infection. The restored ability of GABA sensing and virulence in the complemented strain on tobacco plants further demonstrated the significance of McpG for *Pta*6605. Consistent with the present results, previously studied plant pathogenic bacteria have exhibited chemotaxis abilities to various plant-related compounds (Matilla and Krell, 2018). Adding to the current theory, GABA plays a significant role in plant-microbe communication, either symbiotic or parasitic.

## Citation

Tumewu, S. A., Matsui, H., Yamamoto, M., Noutoshi, Y., Toyoda, K., and Ichinose, Y. (2020) Requirement of γ-Aminobutyric Acid Chemotaxis for Virulence of *Pseudomonas syringae* pv. *tabaci* 6605. *Microbes Environ ***35**: ME20114.

https://doi.org/10.1264/jsme2.ME20114

## Supplementary Material

Supplementary Material

## Figures and Tables

**Table 1.** Bacterial strains and plasmids used in the present study

Bacterial strain, plasmid	Relevant characteristics	Reference or source
*Escherichia coli*		
DH5α	*F^–^λ^–^ ϕ80dLacZ *Δ*M15 *Δ*(lacZYA-argF)U169 recA1 endA1 * *bhsdR17(rK^–^ mK^+^) supE44 thi-1 gyrA relA1*	Nippon Gene
S17-1	*thi pro hsdR hsdR hsdM^+^ recA(chr::RP4-2-Tc::Mu-Km::Tn7)*	Schäfer *et al.*, 1994
*Pseudomonas syringae* pv. *tabaci*		
Isolate 6605	Wild-type isolated from tobacco, Nal^r^	Ichinose *et al.*, 2020
6605-ΔRS26685	Isolate 6605 ΔPS26685 (Δ*mcpG*), Nal^r^	This study
6605-ΔRS06980	Isolate 6605 ΔRS06980, Nal^r^	This study
6605-ΔRS12400	Isolate 6605 ΔRS12400, Nal^r^	This study
6605-Δ*mcpG*-C	pD-*mcpG* containing Δ*mcpG*, Nal^r^ Km^r^	This study
Plasmid		
pGEM-T Easy	Cloning vector, Amp^r^	Promega
pG-RS26685	RS26685 fragment-containing pGEM-TEasy, Amp^r^	This study
pG-RS06980	RS06980 fragment-containing pGEM-TEasy, Amp^r^	This study
pG-RS12400	RS12400 fragment-containing pGEM-TEasy, Amp^r^	This study
pK18*mobsacB*	Small mobilizable vector, Km^r^, sucrose sensitive (s*acB*)	Schäfer *et al.*, 1994
pK18-ΔRS26685	RS26685 deleted DNA-containing pK18*mobsacB*, Km^r^	This study
pK18-ΔRS06980	RS06980 deleted DNA-containing pK18*mobsacB*, Km^r^	This study
pK18-ΔRS12400	RS12400 deleted DNA-containing pK18*mobsacB*, Km^r^	This study
pDSK519	Broad host range cloning vector, Km^r^	Keen *et al.*, 1988
pD-*mcpG*	pDSK519 possessing expressible *mcpG*, Km^r^	This study

Nal^r^, nalidixic acid resistant; Amp^r^, ampicillin resistant; Km^r^, kanamycin resistant.

**Table 2.** Primer sequences used in the present study

Primer Name	Sequence (5′-3′)	Description
RS26685_1	GAGCCCGAAATAACCGAAGA	Amplification of RS26685 and the surrounding region
RS26685_2	CTGGCAATAAACGCGCTGAT	
RS26685_3	GCtctagaTGCCGATAAGGGCCTTTAGA	Deletion of RS26685 ORF
RS26685_4	GCtctagaCAAGCCGCTGCCAGAGAA	
RS06980_1	GTTGCGGCCTTGAAGCTCT	Amplification of RS06980 and the surrounding region
RS06980_2	CCCACGGATGCAGAATAGAC	
RS06980_3	GCtctagaAGACAATATTTTGCCGCACC	Deletion of RS06980 ORF
RS06980_4	GCtctagaCATCCAGTAACAGAGGTCGG	
RS12400_1	GACGATCTTTGGCAGCGGT	Amplification of RS12400 and the surrounding region
RS12400_2	GGAACTGTTTGCTGAGATCC	
RS12400_3	GCtctagaATAGCGGTTACTTCCACGGC	Deletion of RS12400 ORF
RS12400_4	GCtctagaGATGTACAGGTCCCGATGGG	

Lowercase letters indicate the artificial nucleotide sequence for *Xba*I in RS26685_3, RS26685_4, RS06980_3, RS06980_4, RS12400_3, and RS12400_4.
