# Supplementary material for: Requirement of γ-Aminobutyric Acid Chemotaxis for Virulence of Pseudomonas syringae pv. tabaci 6605
Source: Microbes Environ. 2020 Nov 7;35(4):ME20114. doi: 10.1264/jsme2.ME20114 (PMC7734410; doi:10.1264/jsme2.ME20114)
Supplement: Supplementary file 1 — Supplementary Material [file 35_20114_s1.pdf]

Supplemental materials

Fig. S1 Construction of the RS26685, RS06980, and RS12400 mutants. Light gray-shaded arrowheads indicate the position of the PCR primers that amplify each ORF with upstream and downstream regions. Black arrowheads indicate the position of the PCR primers that amplify the upstream and downstream regions prior to ORF deletion.

Fig. S2 Aligned amino acid sequences of *Pseudomonas* strains mentioned in Fig. 1B. Colored backgrounds indicate identical sequence to McpG of *Pta*6605. Cov and pid values indicate percent coverage and percent identity, respectively. Multiple sequence alignment was generated using T-Coffee (tcoffee.crg.cat) (Notredame *et al.* 2000).

Notredame, C., Higgins, D.G. and Heringa J. (2000). T-Coffee: A novel method for fast and accurate multiple sequence alignment. *J Mol Biol* **302**: 205-217.

Fig. S3 Growth of WT and Δ*mcpG* strains in King's B medium/MMMF medium (A) and in MM supplemented with 10 mM GABA as a sole source of carbon and nitrogen (B). Bacterial growth was measured at OD_{595}. Error bars represent standard error from two independent experiments conducted in triplicates.

Fig. S4 Maximum likelihood tree based on a signaling domain (SD) of the amino acid receptors and their homologs in *Pseudomonas* species. Amino acid sequences of SD of McpG and McpA of *P. putida* KT2440, PctA, PctB, and PctC of *P. aeruginosa* PAO1, CtaA, CtaB, and CtaC of *P. fluorescens* Pf0-1, PscA, PscB, and PscC of *Pto*DC3000 and *Psa*NZ-V13, and RS26685, RS12400, and RS06980 of *Pta*6605 were compared. Corresponding ligands are indicated by bold letters (**aa** and **GABA**). Branch length and bootstrap values are indicated on the tree. The tree was generated using MEGA version X software.

Fig. S1

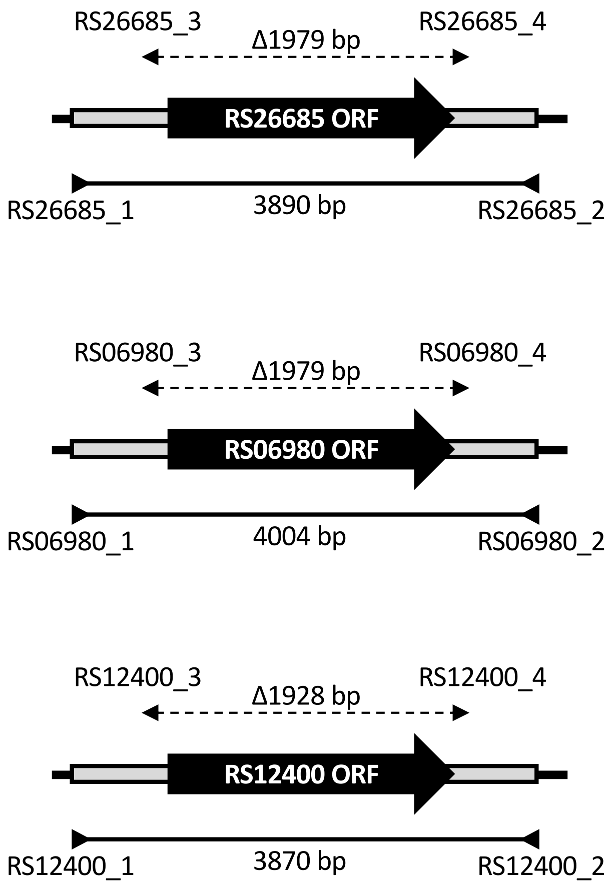
RS26685_3
Δ1979 bp
RS26685_4
RS26685 ORF
RS26685_1
3890 bp
RS26685_2
RS06980_3
Δ1979 bp
RS06980_4
RS06980 ORF
RS06980_1
4004 bp
RS06980_2
RS12400_3
Δ1928 bp
RS12400_4
RS12400 ORF
RS12400_1
3870 bp
RS12400_2

Fig. S2

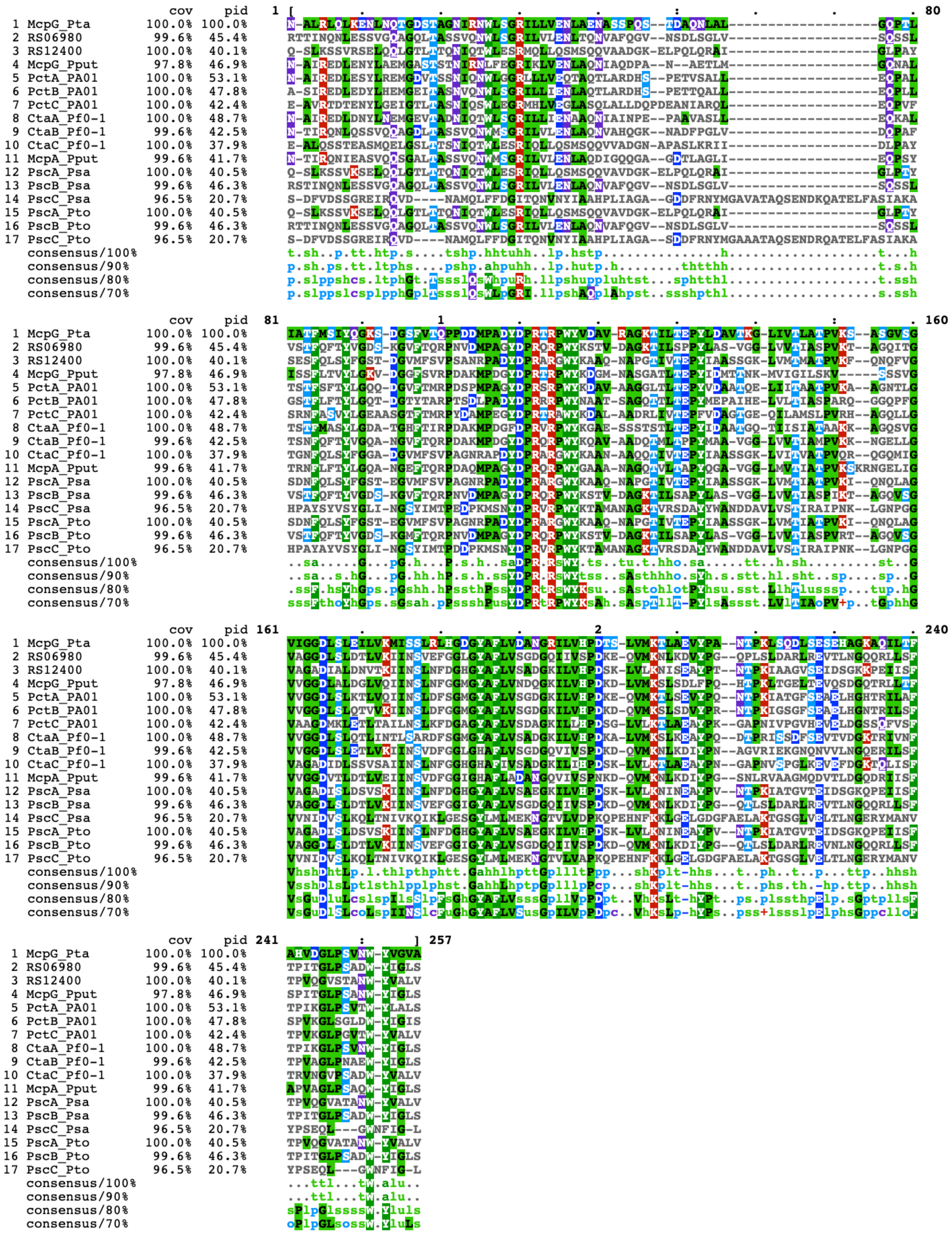
cov pid
1 McpG_Pta 100.0% 100.0%
2 RS06980 99.6% 45.4%
3 RS12400 100.0% 40.1%
4 McpG_Pput 97.8% 46.9%
5 PctA_PA01 100.0% 53.1%
6 PctB_PA01 100.0% 47.8%
7 PctC_PA01 100.0% 42.4%
8 CtaA_Pf0-1 100.0% 48.7%
9 CtaB_Pf0-1 99.6% 42.5%
10 CtaC_Pf0-1 100.0% 37.9%
11 McpA_Pput 99.6% 41.7%
12 PscA_Psa 100.0% 40.5%
13 PscB_Psa 99.6% 46.3%
14 PscC_Psa 96.5% 20.7%
15 PscA_Pto 100.0% 40.5%
16 PscB_Pto 99.6% 46.3%
17 PscC_Pto 96.5% 20.7%
consensus/100%
consensus/90%
consensus/80%
consensus/70%

Fig. S3

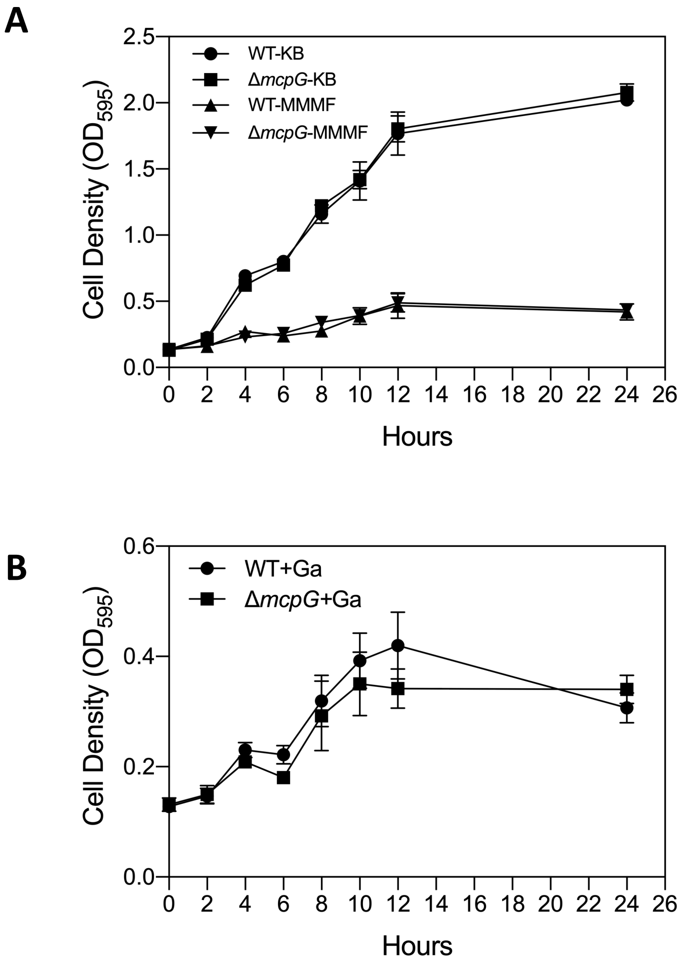
A
WT-KB
ΔmcpG-KB
WT-MMMF
ΔmcpG-MMMF
Cell Density (OD595)
2.5
2.0
1.5
1.0
0.5
0.0
0 2 4 6 8 10 12 14 16 18 20 22 24 26
Hours
B
WT+Ga
ΔmcpG+Ga
Cell Density (OD595)
0.6
0.4
0.2
0.0
0 2 4 6 8 10 12 14 16 18 20 22 24 26
Hours

Fig. S4

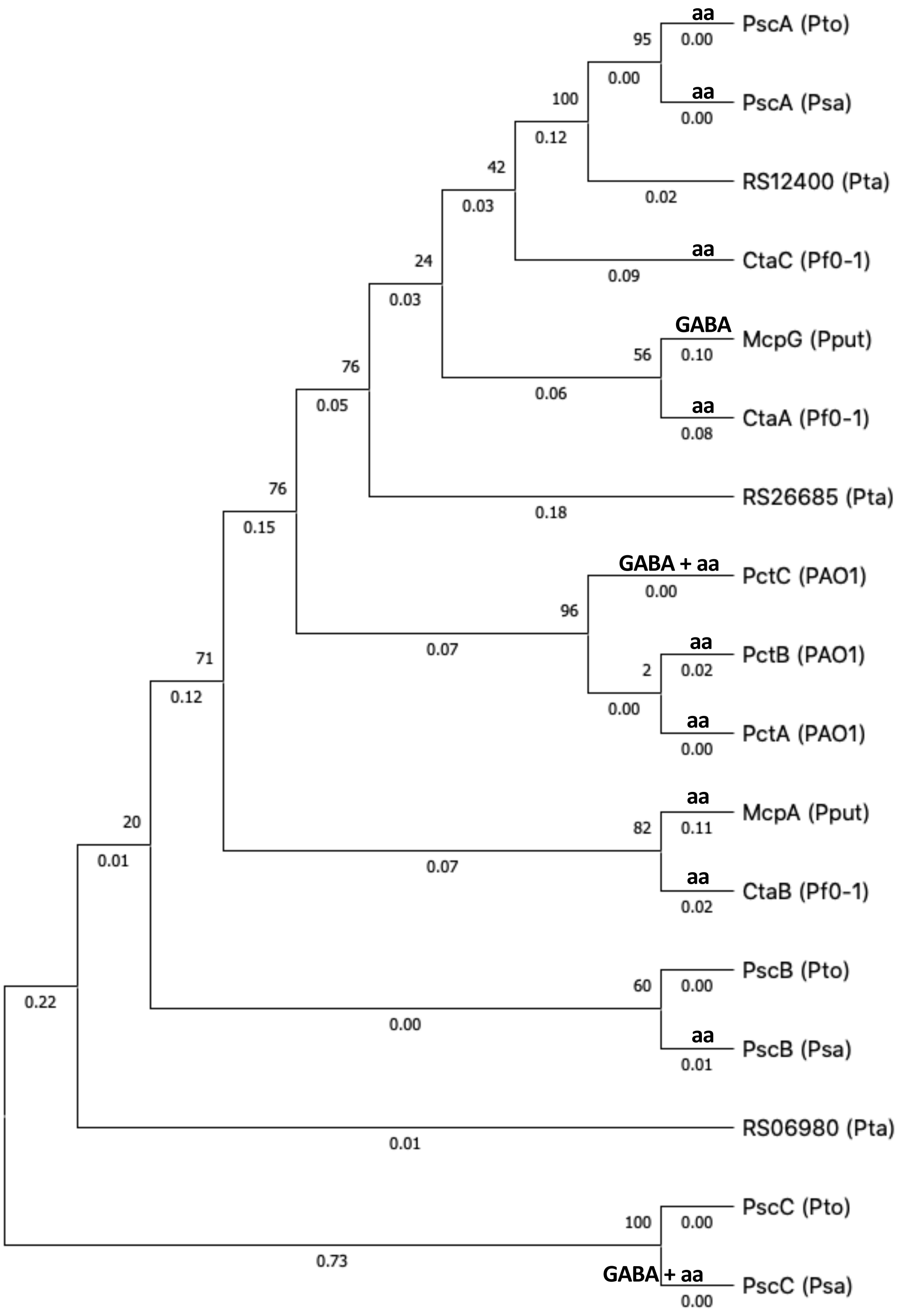

aa
PscA (Pto)
95
0.00
0.00
aa
PscA (Psa)
100
0.00
0.12
RS12400 (Pta)
0.02
42
0.03
aa
CtaC (Pf0-1)
0.09
24
0.03
GABA
McpG (Pput)
56
0.10
0.06
aa
CtaA (Pf0-1)
0.08
76
0.05
RS26685 (Pta)
0.18
76
0.15
GABA + aa
PctC (PAO1)
0.00
96
0.07
aa
PctB (PAO1)
2
0.02
0.00
aa
PctA (PAO1)
0.00
71
0.12
aa
McpA (Pput)
82
0.11
0.07
aa
CtaB (Pf0-1)
0.02
20
0.01
PscB (Pto)
60
0.00
0.00
aa
PscB (Psa)
0.01
0.22
RS06980 (Pta)
0.01
PscC (Pto)
100
0.00
0.73
GABA + aa
PscC (Psa)
0.00
